# Does relationship satisfaction and financial aid from offspring influence the quality of life of older parents?: a longitudinal study based on findings from the Korean longitudinal study of aging, 2006–2012

**DOI:** 10.1186/s12955-016-0509-4

**Published:** 2016-07-26

**Authors:** Yeong Jun Ju, Kyu-Tae Han, Tae-Hoon Lee, Woorim Kim, Juyeong Kim, Eun-Cheol Park

**Affiliations:** 1 Department of Public Health, Graduate School, Yonsei University, Seoul, Republic of Korea; 2 Institute of Health Services Research, Yonsei University, Seoul, Republic of Korea; 3 Department of Preventive Medicine, Yonsei University College of Medicine, Seoul, Republic of Korea; 4 Department of Preventive Medicine and Institute of Health Services Research, Yonsei University College of Medicine, 50 Yonsei-ro, Seodaemun-gu, Seoul, 120-752 Korea

**Keywords:** Quality of life, Older parents, Offspring relationship satisfaction, Offspring support, Marital status

## Abstract

**Background:**

Quality of life (QoL) in old age is of major importance because the global population is aging rapidly. Offspring support, including financial and emotional support, is important in later life and directly affects the wellbeing of elderly individuals. The aim of this study was to examine the relationship between QoL in older parents and offspring support.

**Methods:**

We used baseline data from the 2006–2012 Korean Longitudinal Study of Aging, from 3,274 individuals aged 65 years or older. We measured the individual’s QoL using a visual analog scale and included both relationship satisfaction and regular economic support as variables. A generalized estimating equation (GEE) model was used to perform longitudinal regression analysis on the data.

**Results:**

Regarding the QoL of older parents, those with an unsatisfying relationship with their offspring had a QoL of -21.93 (SE = 0.55; *P* < 0.0001) compared to those with satisfying offspring relationships. Those receiving no regular financial aid from their offspring had a QoL of -0.92 (SE = 0.38; *P =* 0.0171) compared to those who received such economic support. Combination effects were observed, with cases living alone – and having poor offspring relationships and no regular financial support from their offspring – showing the most drastic decrease in QoL (-23.46; SE = 1.03; *P* < 0.0001).

**Conclusions:**

Offspring support influences the QoL of elderly individuals, and Korean children appear to play a crucial role in the QoL of their (older) parents. Considering that the role of offspring is rapidly diminishing due to industrialization policies, initiatives are required to revitalize offspring support for elderly parents.

## Background

Populations around the world are rapidly aging, and the 60 years or older age group accounted for 524 million people worldwide in 2010. That proportion is expected to nearly triple, to reach about 1.5 billion, by 2050 [1]. Because the aging process is associated with increased adverse outcomes such as chronic conditions, disabilities, physical and psychological problems, and quality of life outcomes, it is important for adults to enter later life stages in relatively good health [2–4]. Hence, comprehensive public health initiatives directed toward the aging population are very important, and society is paying greater attention to aging, with respect to physical, mental, and social status, by seeking to enhance quality of life (QoL) among the elderly population [5, 6].

Such concerns are particularly relevant in Korea, which has one of the most rapidly aging populations in the world. The recent increase in the elderly population of Korea has in turn increased concerns related to their psychological wellbeing, expressed for example in QoL. In fact, Koreans are slightly less satisfied with their lives than the Organisation for Economic Cooperation and Development (OECD) average. Based on the Better Life Index, an indicator of QoL, Korea ranked 29^th^ among OECD countries in 2014 (Korea’s score: 5.8/10; OECD average: 6.6/10) [7]. Moreover, Korea has the highest elderly poverty rate among developed countries, and the number of older Korean adults living alone has increased steeply [8]. Considering that low socioeconomic status reduces QoL [9], we can infer that the Korean elderly population faces a lower QoL. In addition, even though Korea has one of the fastest-ageing populations, empirical knowledge regarding the QoL in this population is limited. There are many issues and debates related to the aging society of Korea, but we focused on QoL among the elderly population.

Previous studies showed that poverty is a risk factor for decreased QoL, and also that financial hardship is crucial in determining QoL in the elderly [9, 10]. Consequently, we can infer that guaranteed financial security would improve the QoL of elderly individuals. Few studies have examined how financial support received by an elderly person from their offspring impacts their QoL [11, 12]. Emotional support from offspring is also an important factor influencing QoL among the elderly population [13, 14]. Older parents experience reduced social activity, so their social and emotional relationship with their adult offspring is vital [15]. Meanwhile, one study reported that marital status was associated with QoL [16]. Since the tradition of family care that has played a dominant role in old-age security is currently breaking down due to rapid industrialization and urbanization, interest in this topic has increased in Korea [17]. Thus, it is important to examine the effect of offspring support on QoL among elderly Koreans.

Although previous studies on QoL, as it relates to adult offspring support, have been conducted in various Eastern and Western countries [18, 19], few studies have been performed in Asian countries, including Korea. However, the few studies that have been conducted in Korea focused only on financial or emotional support; furthermore, these studies were cross-sectional in nature [11, 12]. This limits our understating of the multidimensionality of the QoL of elderly populations. Thus, the present study used longitudinal data and focused on not only regular financial aid, but also on relationship satisfaction with adult offspring, as an important factor that affects QoL among elderly individuals.

## Methods

### Study population

For this longitudinal study, we used raw data from the the Korean Longitudinal Study of Aging (KLoSA), conducted between 2006 and 2012 by the Korea Labor Institute. The KLoSA is a panel study based on a nationally representative survey of community-dwelling adults aged 45 years or older. The survey, which has been conducted according to 2-year intervals since 2006, by trained interviewers using computer-assisted personal interviewing methods, addresses economic activity, inocme and wealth status, health behaviors, and other variables related to an aging society. Detailed information on the KLoSA is available on the study website (http://www.kli.re.kr/klosa/en/about/introduce.jsp).

Our sample was restricted to individuals aged 65 years or older. Additionally, any respondents who did not provide data on offspring relationship satisfaction, whether they received regular financial support from offspring, gender, marital status, age, education level, houshold income, wealth, job status, perceived health status, limits on activities of daily living, depressive symptoms, smoking, alcohol intake, freqeuncy of contact with neighbors, or number of chronic diseases, were excluded from the study.

The present study included the 2006, 2008, 2010, and 2012 KLoSA surveys in the analysis, thus yielding a baseline population of 3,274 participants. Regarding the baseline data, the year in which a participant responded to the question regarding offspring relationship satisfaction for the first time was considered as the baseline for that participant. In consideration of the longitudinal study design, participants surveyed only once were excluded. From the baseline assessment onwards, each participant was surveyd at least two, and up to four, times. The average number of measures obtained for each participant was 2.69 ± 0.69.

## Measures

### Overall QoL

Overall QoL was measured based on visual analog scale (VAS) data. The VAS is a useful method for the assessment of QoL [20]. Previous studies used the same VAS to investigate QoL [20–22]. The measure was developed by the Korea Labor Institute and was similar to the EuroQol (EQ-5D) VAS, which asks: “How is your overall quality of life?” Respondents rate their overall QoL on a vertical visual “thermometer” with anchor points of 100 (best imaginable QoL) and 0 (worst imaginable QoL). Despite the limitations of single-item measures, previous studies reported that such measures are generally as reliable and valid as multi-item measures [23, 24].

### Relationship satisfaction with adult offspring

Each individual’s relationship satisfaction with their adult offspring was measured by a single question presented as a vertical VAS. The question was phrased as follows: “How satisfied are you with your relationship with your offspring?” Respondents rated their relationship satisfaction on a scale from 0 to 100, with higher scores corresponding to a higher relationship quality with their offspring. This item was evaluated as a categorical variable according to interquartile range (25 % [quartile 1] to 75 % [quartile 3]). Based on the interquartile range of respondents’ scores, we divided them into the following quartiles: “highly unsatisfied” (<70), “slightly unsatisfied” (< 80), “slightly satisfied” (< 90), and “highly satisfied” (≥ 90). Higher quartiles corresponded to higher relationship quality with adult offspring.

Our question was similar to that measuring the relationship with one’s own children in the Survey of Health, Ageing and Retirement in Europe (SHARE). The reliability and validity of that item are well-established [25].

### Regular financial aid from adult offspring

Regular financial aid from adult offspring was measured by asking the respondents whether they received financial support (monetary or non-monetary transfer) from any of their offspring. Financial aid referred to cash, support for leisure activities, dining out and food, and the provision of health-related products, household items, and electronic equipment. Receipt of regular financial aid was categorized as “yes” if a respondent received financial aid from any of their adult offspring and as “no” if they did not receive such support. Previous studies used the same method to investigate financial aid from adult offspring [11].

### Household income and wealth

Household income was defined as the sum of the incomes of all household members. We divided household income into four equal quartiles (high, middle–high, middle–low, and low). In addition, we calculated household wealth using a previously published method [26, 27]: Net assets = Total assets - Total debts; Total assets = Real-estate assets (present dwelling + other expected present dwellings + land + buildings + fully or partially paid-for lot-solid apartment) + Financial assets (savings + earnings from rental houses + monthly rental income) + Other assets (automobiles + other non-financial assets); and Total debts = Debts (bank loans + individual/loan-company/holding-office loans + rent due) + Deposit for rental property (money paid to secure a lease, to be repaid at some future point).

### Covariates

We included data on age, gender, education level (uneducated, less than middle school, or more than high school), marital status (married or single, including separated, divorced, or widowed), employment status (employed or unemployed), health status (perceived health status [healthy or unhealthy] and limitation on daily activities [yes or no]), health behavior (smoking [yes or no] and alcohol consumption [yes or no], total number of chronic medical conditions (diabetes mellitus, hypertension, cancer and malignant neoplasm, pulmonary disease, liver complaints, cardiovascular disease, and cerebrovascular disease), and depressive symptoms (absent or present). Depressive symptoms were measured by the 10-question Centers for Epidemiologic Studies Depression Scale (CES-10). The “social factor” variable was frequency of contact with neighbors (none, meet once a month or less, or meet more than once a month). The “Living with children” item pertained to whether the respondents lived with their adult offspring; living with one or more such offspring was categorized as “yes;” all other answers were categorized as “no.”

### Statistical analysis

T-tests were used to examine the general baseline characteristics of the study population, and analysis of variance was used to compare mean ± SD QoL scores according to specific characteristics. We evaluated relationships between offspring support and QoL using the generalized estimating equation (GEE) model, which is an extension of the quasi-likelihood approach used to analyze longitudinal correlated data. The GEE model is used to analyzing longitudinal data, as it accounts for time variation and correlations among repeated measurements [28]. All independent variables were adjusted. Subgroup analysis was performed to evaluate possible associations between QoL and offspring support according to marital status. Statistical analyses were performed using the GENMOD procedure in SAS software (ver. 9.4 ;(SAS Institute, Cary, NC, USA). *P*-values were two-sided and considered significant at *P* < 0.05.

## Results

The baseline characteristics of the study population is shown in Table 1. Among the 3,274 individuals, 23.7 % (*n* = 776) reported having a highly unsatisfying relationship with their offspring, whereas 46.4 % (*n* = 1,520) reported having a highly satisfying relationship. The mean baseline QoL scores of those with highly unsatisfactory, slightly unsatisfactory, slightly satisfactory, and highly satisfactory relationships with their adult offspring were 38.63, 53.78, 59.85, and 68.85, respectively. When subjects did receive regular financial aid from their offspring, the mean baseline QoL was higher compared with those who did not receive such support. Regarding marital status, 36.1 % (*n* = 1,182) of respondents were single; the mean baseline QoL of these respondents was lower compared with those who were married.

**Table 1:** General characteristics of the study populations at the baseline

Variables	Baseline					
	Subject		Quality of life			
	N	%	Mean ± S.D			*P*-value
Relationship satisfaction with offspring						<.0001
Highly unsatisfied	776	23.7	38.63	±	20.06	
Slightly unsatisfied	325	9.9	53.78	±	14.93	
Slightly satisfied	653	20.0	59.85	±	15.69	
Highly satisfied	1,520	46.4	68.86	±	17.88	
Regular financial aid from offspring						<.0001
Yes	1,443	44.1	60.90	±	19.73	
No	1,831	55.9	56.43	±	22.55	
Sex						<.0001
Male	1,329	40.6	61.30	±	20.25	
Female	1,945	59.4	56.42	±	22.04	
Age						0.0044
65-69	1,471	44.9	60.03	±	20.62	
70-79	1,393	42.6	57.49	±	22.03	
≥ 80	410	12.5	55.66	±	22.08	
Marital status						0.6635
Married	2,092	63.9	60.79	±	20.22	
Single	1,182	36.1	54.16	±	22.91	
Education level						<.0001
More than high school	553	16.9	66.58	±	19.62	
Less than middle school	1,454	44.4	59.76	±	20.53	
Uneducated	1,267	38.7	53.27	±	21.95	
Household income						<.0001
Q4 (High)	793	24.2	65.08	±	17.26	
Q3	902	27.6	58.65	±	20.15	
Q2	828	25.3	53.62	±	22.11	
Q1 (Low)	751	22.9	56.31	±	24.34	
Wealth						<.0001
Q4 (High)	845	25.8	67.04	±	17.90	
Q3	863	26.4	63.40	±	17.91	
Q2	805	24.6	54.60	±	20.93	
Q1 (Low)	761	23.2	47.16	±	23.41	
Employment status						0.0156
Employed	652	19.9	61.78	±	19.87	
Unemployed	2,622	80.1	57.59	±	21.76	
Living with offspring						0.1164
Yes	1,320	40.3	58.23	±	20.88	
No	1,954	59.7	58.51	±	21.85	
Smoking						0.2098
No	2,787	85.1	58.75	±	21.51	
Yes	487	14.9	56.37	±	21.09	
Alcohol intake						0.0573
No	1,247	38.1	59.39	±	20.28	
Yes	2,027	61.9	57.79	±	22.14	
Perceived health status						<.0001
Healthy	1,824	55.7	64.63	±	18.34	
Unhealthy	1,450	44.3	50.56	±	22.52	
Limits on activities of daily living						0.0003
No	1,758	53.7	63.95	±	18.53	
Yes	1,516	46.3	51.96	±	22.80	
Depressive symptoms						<.0001
No	1,771	54.1	64.60	±	19.11	
Yes	1,503	45.9	51.10	±	21.79	
Frequency of contact with a neighbor						<.0001
None	318	9.7	48.30	±	24.18	
Meet once a month or less	586	17.9	56.83	±	20.58	
Meet once a month or more	2,370	72.4	60.14	±	20.89	
Number of chronic disease						0.0100
None	1,165	35.6	61.54	±	19.74	
1	1,791	54.7	57.87	±	21.83	
2+	318	9.7	49.90	±	22.90	
Total	3,274	100	58.40	±	16.03	

Table 2 lists the associations between offspring support and QoL. After adjusting for covariates, and taking those who had a highly satisfying relationship with offspring as the reference group, participants who had a highly unsatisfying relationship with offspring had a QoL score of -21.93 (SE = 0.55; *P* < 0.0001). Individuals who did not receive regular financial aid from their offspring had a QoL of -0.92 (SE = 0.38; *P* = 0.0171) compared with those who received regular financial aid from their offspring. In addition, low education level, marital status, low income or level of wealth, health status perceived as being unhealthy, depressive symptoms, greater number of chronic diseases, and reduced frequency of contact with neighbor were associated with a lower QoL.

**Table 2:** Results of the GEE analyzing the effects of offspring support

Variables	Quality of life		
	β	S.E	*p*-value
Relationship satisfaction with offspring			
Highly satisfied	Ref.	-	-
Slightly satisfied	-7.26	0.44	<.0001
Slightly unsatisfied	-12.15	0.52	<.0001
Highly unsatisfied	-21.93	0.55	<.0001
Regular financial aid from offspring			
Yes	Ref.	-	-
No	-0.92	0.38	0.0171
Sex			
Male	Ref.	-	-
Female	-0.77	0.53	0.1426
Age			
65-69	Ref.	-	-
70-79	0.39	0.41	0.3426
≥ 80	0.62	0.58	0.2842
Education level			
More than high school	Ref.	-	-
Less than middle school	-0.67	0.65	0.3022
Uneducated	-3.46	0.71	<.0001
Marital status			
Married	Ref.	-	-
Single	-1.34	0.53	0.0112
Household income			
Q4 (High)	Ref.	-	-
Q3	-1.91	0.47	<.0001
Q2	-4.06	0.52	<.0001
Q1 (Low)	-3.61	0.56	<.0001
Wealth			
Q4 (High)	Ref.	-	-
Q3	-2.05	0.45	<.0001
Q2	-5.11	0.48	<.0001
Q1 (Low)	-9.20	0.54	<.0001
Employment status			
Employed	Ref.	-	-
Unemployed	-0.51	0.45	0.2570
Living with offspring			
Yes	Ref.	-	-
No	1.46	0.43	0.0007
Smoking			
No	Ref.	-	-
Yes	-1.21	0.55	0.0284
Alcohol intake			
No	Ref.	-	-
Yes	0.24	0.46	0.5991
Perceived health status			
Healthy	Ref.	-	-
Unhealthy	-5.78	0.41	<.0001
Limits on activities of daily living			
No	Ref.	-	-
Yes	-2.49	0.39	<.0001
Depressive symptoms			
No	Ref.	-	-
Yes	-3.81	0.36	<.0001
Frequency of contact with a neighbor			
None	Ref.	-	-
Meet once a month or less	-2.02	0.62	0.0011
Meet once a month or more	-0.16	0.44	0.7162
Number of chronic disease			
None	Ref.	-	-
1	-0.56	0.41	0.1664
2+	-2.21	0.66	0.0008
Survey year			
2006	Ref.	-	-
2008	0.53	0.41	0.2048
2010	2.49	0.61	<.0001
2012	1.35	0.63	0.0341

Notes: Adjusting for retirement satisfaction, regular financial aid from offspring, sex, age, education level, marital status, household income, wealth, employment status, living with offspring, smoking, alcohol intake, perceived health status, limits on activities of daily living, depressive symptoms, frequency of contact with a neighbor, number of chronic disease, survey year

**Fig. 1:**
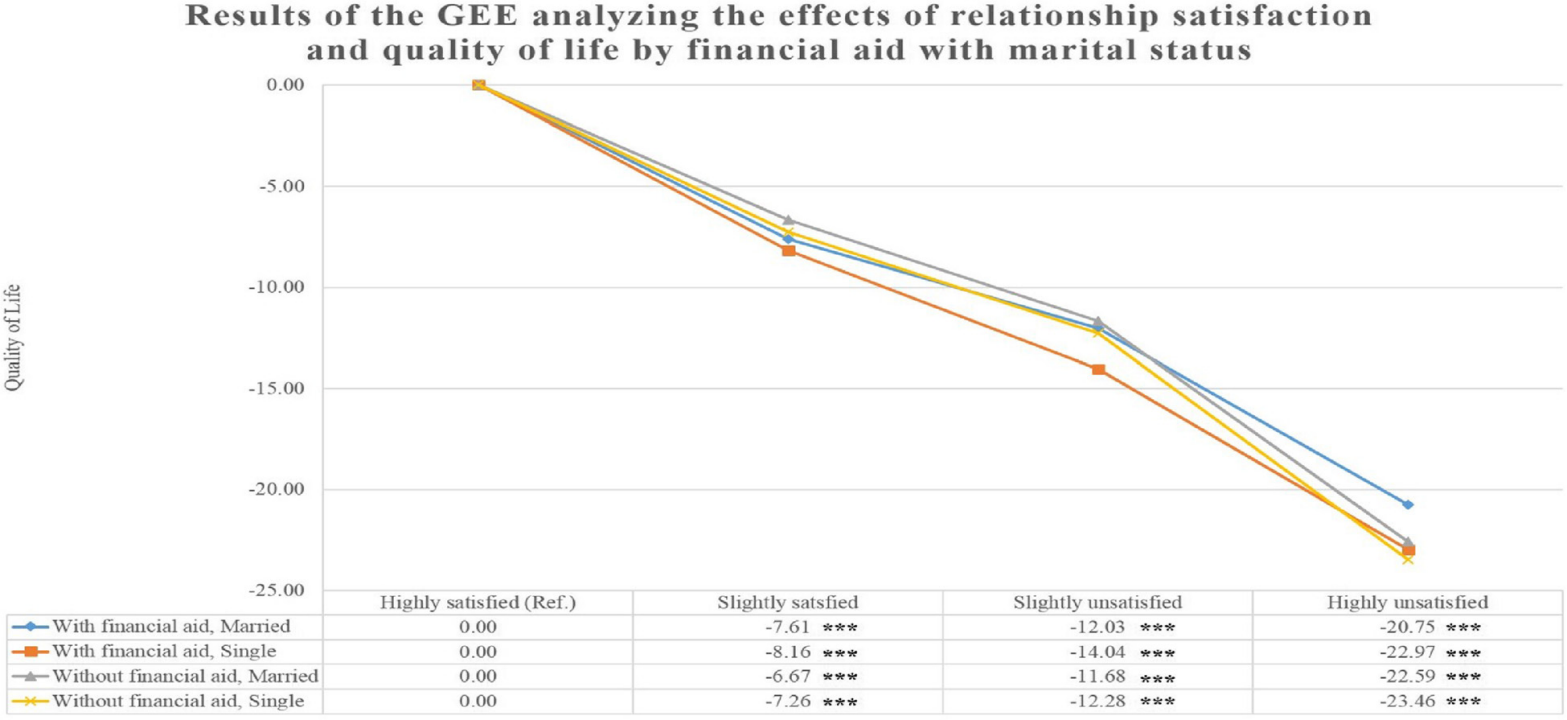
Adjusted effects of relationship satisfaction and QoL by financial aid with marital status. Adjusted for sex, age, education level, marital status, household income, wealth, employment status, living with offspring, smoking, alcohol intake, perceived health status, limits on activities of daily living, depressive symptoms, frequency of contact with a neighbor, number of chronic disease, survey year. *** *P* < 0.001

Figure 1 shows associations between relationship satisfaction and QoL by financial aid with marital status. Individuals with a highly unsatisfying relationship with offspring, a lack of financial aid from offspring, and those that lived alone showed the most drastic decrease in QoL (-23.46; SE = 1.03; *P* < 0,0001).

## Discussion

The recent increase in the elderly population of Korea has in turn increased concerns related to social issues, such as poverty [29]. In fact, Korea has the highest poverty rate among elderly individuals, with one-half of Koreans aged 65 years or older living in relative poverty (poverty rate = 49.6 % in Korea vs. 12.6 % among the other OECD countries) [30]. Additionally, the number of older Korean adults living alone has increased 2.5-fold since 2000, and currently accounts for a quarter of the elderly population [8]. These phenomena have intensified in recent years, because the tradition of family care, including offspring support, which has played a dominant role in old-age security is rapidly breaking down due to modernization and industrialization [11, 12]. This indicates that the overall wellbeing (i.e., QoL) of older adults may be at risk. Thus, it is important to design effective strategies to improve QoL among elderly populations.

We found that offspring support (relationship satisfaction, regular financial aid) influenced QoL among our elderly sample. Our results are similar to previous findings indicating that low relationship satisfaction with, and lack of financial aid from, offspring contributed to a lower QoL. Furthermore, QoL estimates were more significantly associated with relationship satisfaction with, versus regular financial aid from, adult offspring. These findings are similar to those of previous reports. In previous studies, emotional support was the highest dimension of offspring supports [31], and adult children who enjoy good relationships with their parents tend to provide more help and support [32, 33]. Our subgroup analysis confirmed that offspring support was associated with QoL among living alone individuals without a spouse and lack of financial support. Elderly individuals with living alone and lack of financial support in particular may be more vulnerable to the financial security compared with who did not. Moreover, elderly individuals with financial insecurity and unsatisfactory relationship with offspring may experience deteriorated psychological wellbeing, in particular, QoL.

Our findings indicate that filial duty still plays a crucial role in the QoL of older parents. As family care is rapidly breaking down due to rapid industrialization, the elderly Korean population encounters difficulties in later life. In addition, whereas Western countries have adopted a social pension scheme to support its aging population, the Korea national pension scheme does not guarantee later life security for elderly individuals becuase of immature pension programme and limited government welfare spending [34]. Thus, it is important to address this issue, as well as QoL, in the elderly population.

Several important insights relevant to improving QOL among the retired elderly population emerged from our data. First, interventions focused on developing both satisfying emotional and material relationships between adult offspring and their older parents may be particularly important in terms of improving QoL in the latter group. One method that may improve the satisfaction derived from an emotional relationship between adult offspring and their older parents is improvement of the work environment. According to OECD statistics, Korea has the second longest average working week in the world, of 44 hours. Considering these excessive work hours, the number of hours that an adult child spends with their parents may be expected to decrease accordingly. Therefore, companies – as well as the government – should improve the work environment to allow for more contact between adult children and their parents. Additionally, to ensure financial security among the elderly, the government should encourage adult offspring to live with their parents, thereby enhancing their financial security. In addition, the government should focus on improving the national pensions of elderly individuals to guarantee that these individuals can attain financial security. Finally, the ultimate goal is restoration of family solidarity, because this variable strongly affects the wellbeing of older adults [35].

## Strengths and limitations

This study had some strengths compared to previous studies. First, it used a large, representative sample, as well as a longitudinal design, with data collected from nationally representative populations. Second, to the best of our knowledge, this study is the first to report on the relationship between the QoL of older parents and the degree of support that they receive from their offspring, in terms of the combination between offspring relationship satisfaction and marital status. In addition, previous studies focused on financial support or emotional support alone, while the present study takes into account both factors. Finally, our study focused on the determinants of QoL among the elderly population.

However, the present study also had several limitations. First, there may be measurement errors associated with the respondents’ subjective responses. To more accurately illustrate the relationship between level of offspring support and QoL in adult parents, other objective tools must be considered. Second, the causal relationship between the independent variables (economic support from, and relationship satisfaction with, offspring) and the dependent variable (QoL) was tested in only one direction, such that the results could have reflected reverse causality. Therefore, we cannot rule out that a lower QoL causes a decrease in offspring support, or whether there is some other explanation. Third, further studies are required to examine QoL using multiple measurements.

## Conclusions

There was a significant relationship between QoL in older parents and their level of offspring support. Our results suggest that offspring support plays an important role in improving overall wellbeing among the elderly population. Thus, institutional support, and policies promoting filial duty, should be implemented.

## Abbreviations

QoL, Quality of Life; OECD, Organization for Economic Co-operation and Development; KLoSA, Korean Longitudinal Study of Aging; VAS, Visual analogue scales; GEE, Generalized estimating equations; S.E, standard error
